# Usefulness of Geriatric Parameters in Preoperative Evaluation of Patients Undergoing Minimally Invasive Surgery for Endometrial Cancer: A Retrospective Cohort Study

**DOI:** 10.1245/s10434-025-17376-9

**Published:** 2025-05-16

**Authors:** Jonas Jean Mathieu Vibert, Franziska Siegenthaler, Flurina A. M. Saner, Stefan Mohr, Michael D. Mueller, Sara Imboden

**Affiliations:** Department of Obstetrics and Gynecology, Bern University Hospital, University of Bern, Bern, Switzerland

**Keywords:** ACCI, EMCA, Sentinel lymph node dissection, Radical lymphadenectomy, Perioperative morbidity, Survival analysis

## Abstract

**Introduction:**

Patients operated for endometrial cancer (EMCA) are typically elderly with multiple comorbidities, potentially impacting surgical outcomes and survival. This study evaluated the prognostic value of frailty and frailty-related scores in predicting perioperative morbidity and survival in EMCA patients undergoing minimally invasive surgery.

**Methods:**

This retrospective cohort study included 289 patients from the Sentinel Database treated for EMCA at Bern University Hospital (2012–2020). Patients underwent minimally invasive hysterectomy with sentinel lymph node dissection (39%) or additional radical lymphadenectomy (61%). Frailty was assessed using the Age-Adjusted Charlson Comorbidity Index (ACCI), modified Frailty Index (mFI), 5-item mFI (mFI-5), American Society of Anesthesiologists (ASA) scores, and independent parameters. Primary outcomes included perioperative complications, hospital stay, recurrence-free survival (RFS), and overall survival (OS).

**Results:**

Median age was 65 years (range 26–94) and median follow-up was 41 months (0–105). ACCI > 4 (23.2%) was the strongest predictor of postoperative complications (*p* = 0.025), prolonged hospitalization (*p* = 0.03), and reduced OS (hazard ratio [HR] 2.57, 95% confidence interval [CI] 1.18–5.60; *p* = 0.018). Multivariable analysis confirmed ACCI > 4 (HR 2.24, 95% CI 1.02–4.90; *p* = 0.044), European Society for Medical Oncology (ESMO) risk group (HR 1.61, 95% CI 1.24–2.07; *p* < 0.001), hemoglobin (HR 1.03, 95% CI 1.00–1.05; *p* = 0.033), and congestive heart failure (HR 6.29, 95% CI 1.35–29.27; *p* =0.019) were significant predictors of OS. Radical lymphadenectomy (*p* < 0.001), ACCI > 4 (*p* = 0.025), and age > 70 years (*p* = 0.034) increased complication risks.

**Conclusions:**

ACCI > 4 is a practical tool for preoperative risk assessment and predicting surgical tolerance and survival, and is therefore applicable for guiding surgical decisions and personalized care in patients with EMCA.

Endometrial cancer (EMCA) is the most common gynecologic cancer among women in developed countries. In most cases (75%), the diagnosis can be made at an early stage following postmenopausal bleeding.^1^ The primary treatment and staging are performed surgically. The role of lymph node dissection (LND) has been a topic of ongoing discussion for decades. The sentinel lymph node (SLN) technique was introduced in the first prospective multicenter study (SENTI-ENDO) in 2011,^2^ and since then it has been increasingly integrated into other guidelines (National Comprehensive Cancer Network [NCCN], S3 Leitlinien, European Society of Gynaecological Oncology [ESGO]). It is expected that in the future, radical LND will be replaced by sentinel LND only, or by anatomic LND^3^; however, this is still under discussion and requires further investigation in large prospective studies. Surgeries with radical LND can lead to significant morbidity and even increased mortality^4^; therefore, a highly personalized medicine is needed to select patients who will profit from the radical surgery, while identifying others where the risk of morbidity following radical surgery might be too high.

As EMCA is a disease affecting predominantly older women, patients with EMCA often present with comorbidities and age-related physiological decline that can complicate treatment decisions. The impact of frailty on surgical outcomes, chemotherapy tolerance, and postoperative recovery in these patients remains an area of growing concern. Identifying frail individuals early in their treatment trajectory may guide personalized therapeutic strategies, such as adjusting the degree of surgery or adjuvant therapies and improving supportive care measures.

Frailty syndrome is a condition often linked to advanced age, although not exclusively. It is defined as a loss of physiologic reserve needed to maintain or regain homeostasis in the face of a stressor.^5^ In concrete terms, it is of relevance to people who are at increased risk of loss of their independence that continues up to mortality. The concept has been recognized for many years, but there was no consensus regarding its definition. It was used to describe a person who seemed to be weak and with an evident fragility. A consensus about its definition was first established in 2013, which defined frailty as *“a medical syndrome with multiple causes and contributors that is characterized by diminished strength, endurance, and reduced physiologic function that increases an individual’s vulnerability for developing increased dependency and/or death”*.^6^

Historically, two main approaches were explored to detect frailty: the clinical phenotype (Fried et al.^7^) and the number of deficits accumulated over time (Mitnitski et al.^8^). Fried et al. used five items to define and detect frailty, i.e. low grip strength, slow walk, low activity, weight loss, and exhaustion, whereas Mitnitski et al. used the history of the patient (comorbidities, cognitive and physical impairments, and psychosocial risk factors) to detect frailty.^9^

Since the syndrome is now recognized as an entity in itself, many different tools have been developed to identify a frail patient. The Comprehensive Geriatric Assessment (CGA)^10^ is considered the gold standard for setting the stage for the management of frailty; however, it is, as are most of the existing tools, time-consuming to apply and not always feasible as an everyday tool in a surgical center. Many simplified tools have been created and analyzed in different contexts; however, to our knowledge, none have been used to assess a patient’s ability to undergo sentinel LND as opposed to radical LND in EMCA.

Since a number of studies showed a clear relation between frailty and poor surgical outcome and/or mortality, including in the field of gynecology,^11–14^ there is a clear need to develop a simple tool that can identify patients at risk of poor EMCA surgical outcome. Such a tool will help the physician determine the radicality of the surgery.

The aim of our study was to examine the prognostic role of frailty scores and other clinical factors on peri- and postoperative morbidity in patients with EMCA, comparing sentinel LND versus radical LND, as well as assessing general oncologic outcome.

## Methods

### Study Design and Participants

We conducted a cohort study on patients from the Sentinel Database, a database collecting surgical data on patients with EMCA who were operated at the Bern University Hospital (Inselspital, Switzerland) during an 8-year period (December 2012–May 2020).

Indocyanine green (ICG) SLN has been performed at our center since December 2012. All data on operations, such as staging and outcomes, were collected prospectively. Further information on demographic, social, and past medical history were obtained from the patients’ electronic health records. Information on each patient’s personal situation (falls during the past year, medication, help at home, auxiliary means of support) was obtained by standardized questionnaires, which were completed for all incoming patients. Follow-up information was obtained from the oncological database updated yearly in the context of the certification of our hospital as a cancer center. Informed consent was obtained from all patients and the study was approved by the Ethics Commission of the Canton of Bern (2018–00479).

All patients underwent laparoscopic total hysterectomy with bilateral salpingo-oophorectomy with either sentinel only or sentinel with radical pelvic and para-aortic lymphadenectomy. The choice between the two surgical approaches was based on the European Society for Medical Oncology (ESMO) risk classification^15^ (information obtained from curettage diagnosis and frozen section of the uterus) and the surgeon’s indication. SLN mapping was performed intraoperatively in all patients using ICG injection in the cervix.

### Independent Variables and Scores

Clinical and demographic data analyzed included type of lymphadenectomy (sentinel vs. radical), stage, grade, lymphovascular space invasion (LVSI), histology, number of positive lymph nodes (LNs+), ESMO risk, follow-up time, recurrence, time to recurrence, vital status, time to death, age, body mass index (BMI), functional status (an individual’s ability to perform normal daily activities required to meet basic needs), number of medications, help with intake of medication, help at home, auxiliary means (none, cane, walker, wheelchair), falls over the past year, weight loss (> 5% in 1 year), American Society of Anesthesiologists (ASA) score, presence of osteoporosis, and laboratory results obtained before the intervention (hemoglobin, total calcium, albumin, and estimated glomerular filtration rate [eGFR]).

### Frailty Scores

#### Age-Adjusted Charlson Comorbidity Index (ACCI)

This score was developed to predict mortality in emergency settings. It initially consisted of 19 indicators (diabetes mellitus with end-organ damage, diabetes mellitus without end-organ damage, cerebrovascular disease, myocardial infarction, congestive heart failure, peripheral vascular disease, dementia, chronic pulmonary disease, connective tissue disease, peptic ulcer disease, mild liver disease, moderate/severe liver disease, moderate/severe renal disease, hemiplegia, solid tumor without metastasis, leukemia, lymphoma, metastatic solid tumor, and AIDS). Patient age, which is an important prognostic parameter to predict survival, was later added to the initial score to create the Age-Adjusted Charlson Comorbidity Index (ACCI) used in the present study.^16^ Its validity has been demonstrated in a number of surgical and oncological situations.^17,18^ Several studies confirm its capacity to predict overall survival (OS; months from surgery to death) in gynecologic cancers^19^ and to predict complications^14,20^; however, to our knowledge, the score has never been used to help determine the extent of the surgical staging.

#### The Modified Frailty Index (mFI)

In 2007, a frailty index based on the 70 items in the Canadian Study of Health and Aging (CSHA) was created.^21^ Eleven variables underpinning the CSHA frailty index were selected by Rubinfeld et al. to create the modified Frailty Index (mFI),^22^ with the latter being validated in surgical and oncologic settings in a number of studies.^12,13,21,23^ The 11 items included in the score were: (1) chronic obstructive pulmonary disease (COPD) or recent pneumonia; (2) congestive heart failure; (3) myocardial infarction; (4) percutaneous coronary intervention (PCI), prior cardiac surgery (PCS), or angina; (5) diabetes mellitus; (6) hypertension requiring medication; (7) peripheral vascular disease or ischemic rest pain; (8) impaired sensorium; (9) transient ischemic attack (TIA) or cerebrovascular accident (CVA); (10) CVA with neurological deficit; and (11) dependent functional health status (total or partial) at time of surgery (30 days prior to surgery). The score was calculated by taking the presence (1 point) or absence (0 points) of the above-mentioned items and then dividing by the total number of items in the score (11). The final score ranged from 0 to 1.0. A cut-off for increased risk of frailty, validated by the results of several studies, was set at > 0.27.^21^

#### The Modified 5-Item Frailty Index (mFI-5)

The 5-item mFI (mFI-5) was created on the basis of the mFI. The selection of five items showed an equivalent predictive capacity as the mFI.^24^ In 2022, Wainger et al. confirmed its capacity to detect patients at potential risk of complications prior to abdominal hysterectomy.^25^ The items selected for this tool were: (1) congestive heart failure; (2) diabetes mellitus; (3) COPD or recent pneumonia; (4) dependent functional health status (total or partial) at time of surgery (30 days prior to surgery); and (5) hypertension requiring medication. The score was obtained by adding up the five individual scores (1 point per item if present; 0 points if absent) and then grouping the scores obtained into three categories (0 points, 1 point, 2–5 points).

### Postoperative Morbidity and Oncological Outcome

Postoperative complications, length of hospital stay, and survival were analyzed. Complications were classified in accordance with the Clavien–Dindo classification^26^; complications that occurred within 30 days after surgery were included. We did not evaluate grade 1 complications, since they strongly depend on the clinician’s evaluation and documentation and have little clinical impact. Oncologic follow-up data collected consisted of OS, recurrence-free survival (RFS; months from surgery to recurrence), and disease-specific survival (DSS), as well as months from surgery to death due to disease.

To fulfill the aim of this study, we compared the role of the frailty scores and other clinical factors in predicting surgical morbidity (complications, hospital stay) and oncologic outcome.

### Statistical Analysis

The significance of the correlation between scores and outcomes was determined using Fisher’s exact test or the Chi-square test, as well as univariate and multivariable analysis. OS, RFS, and DSS were assessed using Cox’s proportional hazard model to perform univariate and multivariable survival analyses and to establish the Kaplan–Meier estimate. *P*-values <0.05 were considered significant. The statistical analysis was performed using SPSS version 28.0.1.0 (IBM Corporation, Armonk, NY, USA).

## Results

A total of 289 patients were included in this study. The cohort consisted of 112 women (39%) who underwent total hysterectomy with SLN dissection only and 177 women (61%) who underwent total hysterectomy with both sentinel LND and radical LND. The median age was 65 years (range 26–94), and the median follow-up period was 41 months (range 0–105). Patient and tumor characteristics are summarized in Table 1, with a comparison of the sentinel LND and radical LND groups.

**Table 1:** Clinical and tumor characteristics and frailty scores by surgical approach

		Sentinel LND	Radical LND	Total	*p*-Value
Patients (*N* = 289)		177	112	289	
Median age, years (range)		64 (26–94)	67 (38–88)	65 (26–94)	0.041
Median BMI, kg/m^2^ (range)		28 (13–57)	27 (18–49)	28 (13–57)	0.035
FIGO stage	Ia Ib II IIIa IIIC1 IIIC2 IVb	134 (75.7) 27 (15.3) 6 (3.4) 6 (3.4) 2 (1.1) 0 (0) 2 (1.1)	36 (32.1) 34 (30.4) 7 (6.25) 6 (5.4) 13 (11.6) 16 (14.3) 0 (0)	270 (58.8) 61 (21.1) 13 (4.5) 12 (4.2) 15 (5.2) 16 (5.5) 2 (0.7)	< 0.001
Histology	Endometrioid Serous Clear cell Mucinous Carcinosarcoma	171 (96.6) 1 (0.6) 0 (0) 2 (1.1) 2 (1.1)	77 (68.75) 15 (13.4) 6 (5.4) 0 (0) 10 (8.9)	248 (85.8) 16 (5.6) 6 (2.1) 2 (0.7) 12 (4.2)	< 0.001
Grade	1 2 3	98 (55.4) 66 (37.3) 13 (7.3)	12 (10.7) 29 (25.9) 71 (63.4)	110 (38.1) 95 (32.9) 84 (29.1)	< 0.001
Lymphovascular space invasion	Yes No	163 (92.1) 14 (7.9)	76 (67.9) 36 (32.1)	239 (82.7) 50 (17.3)	< 0.001
Myometrial infiltration	< 50% > 50%	140 (79.1) 37 (20.9)	57 (50.9) 54 (49.1)	197 (68.2) 91 (31.5)	< 0.001
Cervical stromal invasion	Yes No	6 (3.4) 171 (96.6)	7 (6.3) 105 (93.7)	13 (4.5) 276 (95.5)	0.255
ESMO risk	Low Intermediate High–intermediate High Advanced metastatic	126 (71.2) 27 (15.3) 14 (7.9) 5 (2.8) 5(2.8)	8 (7.1) 26 (23.2) 34 (30.4) 19 (17.0) 25 (22.3)	134 (46.4) 53 (18.3) 48 (16.6) 24 (8.3) 30 (10.4)	< 0.001
Lymph node metastases	Yes No	3 (1.7) 174 (98.3)	25 (22.3) 87 (77.7)	28 (9.7) 261 (90.3)	< 0.001
Adjuvant therapy	Yes No	42 (23.7) 135 (76.3)	99 (88.4) 13 (11.6)	141 (48.8) 148 (51.2)	< 0.001
Follow-up (months)		39	45	41	0.034
Recurrence	Yes No	10 (5.6) 167 (94.4)	17 (15.2) 95 (84.2)	27 (9.3) 262 (90.6)	0.006
Death	Disease Other cause with evidence of disease Other cause without evidence of disease Total	5 (2.8) 1 (0.6) 6 (3.4) 12 (6.8)	10 (8.9) 1 (0.9) 4 (3.6) 15 (13.4)	15 (5.2) 2 (0.7) 10 (3.5) 27 (9.3)	0.454 0.743 0.594 0.06

Data are expressed as *n* (%) unless otherwise specified

*LND* lymph node dissection, *BMI* body mass index, *FIGO* International Federation of Gynaecology and Obstetrics, *ESMO* European Society for Medical Oncology

### Patient Characteristics

The median age of patients differed significantly between the sentinel LND and radical LND groups, with median ages of 64 and 67 years, respectively (*p* = 0.041). BMI was significantly higher in the sentinel LND group (28 vs. 27; *p* = 0.035).

In terms of International Federation of Gynaecology and Obstetrics (FIGO) staging, the majority of sentinel LND patients were stage IA (75.7%), while radical LND patients had a broader distribution, with 32.1% in stage IA and 14.3% in stage IIIC2 (*p* < 0.001). Histological subtypes differed significantly, with 96.6% of sentinel LND patients having endometrioid carcinoma compared with 68.7% in the radical LND group, which had a higher proportion of serous carcinoma patients (13.4%) [*p* < 0.001]. Grade 1 tumors were more common in the sentinel LND group (55.4%), while grade 3 tumors were more frequent in the radical LND group (63.4%) [*p* < 0.001]. LVSI was significantly more prevalent in the sentinel LND group (92.1%) than in the radical LND group (67.9%) [*p* < 0.001]. Similarly, myometrial infiltration of <50% was more common in sentinel LND patients (79.1%) compared with radical LND patients (50.9%) [*p* < 0.001]. There was no significant difference in cervical stromal invasion (*p* = 0.255). Regarding ESMO risk classification, the majority of sentinel LND patients fell into the low-risk category (71.2%), while the radical LND group had a more varied risk profile, with 30.4% in the high–intermediate-risk and high-risk categories (*p* < 0.001). Lymph node metastases were significantly more frequent in the radical LND group (22.3%) compared with the sentinel LND group (1.7%) [*p* < 0.001]. Adjuvant therapy was also more commonly administered in the radical LND group (88.4%) compared with the sentinel LND group (23.7%) [*p* < 0.001].

### Distribution and Cut-Off of the Scores (ACCI, mFI, mFI-5, and American Society of Anesthesiologists Score)

The ASA and ACCI scores were similar across groups, with a median ASA score of 2 (range 1–4) and an ACCI score of 3 (range 0–11). Additionally, no significant differences were found in mFI and mFI-5 scores, with a median mFI score of 0 (range 0–0.45) and an mFI-5 score of 0 (range 0–3) in both groups. Frailty based on mFI > 0.27 and mFI-5 > 1 was observed in 7 patients (2.4%) and 40 patients (13.8%), respectively, with no significant difference between the groups.

In the entire cohort, the ACCI ranged from 0 to 11, with a mean of 3.57. The optimal cut-off for the ACCI, determined by receiver operating characteristic (ROC) curve analysis, was > 4, classifying 67 patients (23.2%) as frail. The mFI ranged from 0 to 0.45, with a mean of 0.07; applying the widely validated cut-off of 0.27, 7 patients (2.4%) were identified as frail. The mFI-5 ranged from 0 to 3, with a median of 0.61; using a cut-off of > 1, 40 patients (13.9%) were classified as frail. The ASA score ranged from 1 to 4, with a mean of 2.4; using cut-offs of > 3 and > 2, 7 patients (2.42%) and 123 patients (42.6%) were classified as frail, respectively. These values are also presented in Table 2, comparing the two groups.

**Table 2:** Comparison of frailty scores between the sentinel and radical LND groups

		Sentinel LND	Radical LND	Total	*p*-Value
ASA score	Median (range)	2 (1–4)	2 (1–4)	2 (1–4)	0.907
ACCI	Median (range)	3 (0–11)	3 (0–9)	3 (0–11)	0.126
mFI	Median (range)	0 (0–0.45)	0 (0–0.36)	0 (0–0.45)	0.222
mFI-5	Median (range)	0 (0–3)	0 (0–3)	0 (0–3)	0.138
ASA (> 2)	In numbers (%)	76 (42.9)	47 (42.0)	123 (42.6)	0.907
ACCI (> 4)	In numbers (%)	40 (22.6)	27 (24.1)	67 (23.2)	0.736
mFI (> 0.27)	In numbers (%)	6 (3.4)	1 (0.9)	7 (2.4)	0.182
mFI-5 (> 1)	In numbers (%)	29 (16.4)	11 (9.8)	40 (13.8)	0.122

*LND* lymph node dissection, *ASA* American Society of Anesthesiologists, *ACCI* Age-Adjusted Charlson Comorbidity Index, *mFI* modified Frailty Index, *mFI-5* 5-item modified Frailty Index

### Other Clinical Variables

The number of medications, help with medication intake, assistance at home, and the use of auxiliary support (e.g., cane, walker, wheelchair) did not differ significantly between the two groups. Falls within the past year and weight loss of > 5% were also comparable (Table 3).

**Table 3:** Comparison of other variables related to frailty between the sentinel and radical LND groups

		Sentinel LND	Radical LND	Total	*p*-Value
Patients (*N* = 289)		177	112	289	
Number of medications		1 (0–15)	2 (0–13)	1 (0–15)	0.776
Help with medication intake	Yes No	11 (6.2) 156 (88.1)	6 (5.4) 99 (88.4)	17 (5.9) 255 (88.2)	0.773
Help at home	Yes No	11 (6.2) 154 (87.0)	5 (4.5) 100 (89.3)	16 (5.5) 154 (53.3)	0.520
Auxiliary support	None Cane Walker Wheelchair	140 (79.1) 6 (3.4) 2 (1.1) 3 (1.7)	88 (78.6) 5 (4.5) 3 (2.7) 0 (0)	228 (78.9) 11 (3.8) 5 (1.7) 3 (1.0)	0.855
Fall over the past year	Yes No	9 (5.1) 82 (46.3)	6 (5.4) 54 (48.2)	15 (5.2) 136 (47.1)	0.983
Loss of weight > 5% in 1 year	Yes No	13 (7.3) 51 (28.8)	10 (8.9) 33 (29.5)	23 (8.0) 84 (29.1)	0.719

Data are expressed as *n* (%) unless otherwise specified

*LND* lymph node dissection

### Postoperative Morbidity

The median length of hospitalization differed significantly between the SLN dissection group (3 days, range 1–12) and the radical LND group (5 days, range 2–42; *p* < 0.001). The mFI (*p* = 0.558) and mFI-5 > 1 (*p* = 0.310) did not predict length of hospitalization, whereas ACCI > 4 (*p* = 0.03) and ASA > 2 (*p* = 0.022) were significant predictors. Length of hospitalization also correlated with patient age (*p* < 0.001).

Postoperative complications classified as Clavien–Dindo grade > 2 (CDI > 2) were observed in 2 patients (1.14%) who underwent sentinel LND and 11 patients (9.8%) who underwent radical LND. Despite the small sample size (*N* = 13), a significant correlation was observed, with *p* < 0.001. Postoperative complications classified as Clavien–Dindo grade > 1 (CDI > 1) were observed in 17 patients (9.6%) who underwent sentinel LND and 31 patients (27.7%) who underwent radical LND (*p* < 0.001). Complications were significantly associated with age (patients over 70 years of age) [*p* = 0.034] and chronic pulmonary disease (*p* = 0.013). A significant association was identified between ACCI > 4 and complications defined as CDI > 2, with patients having ACCI > 4 showing 3.53 times higher odds of developing CDI > 2 (95% confidence interval [CI] 1.10–11.32; *p* = 0.025). Furthermore, complications were significantly associated with the type of surgery, both for CDI > 1 and CDI > 2. CDI > 1 was observed in 17 patients (9.6%) in the sentinel LND group compared with 31 patients (27.7%) in the radical LND group (*p* < 0.001). Similarly, CDI > 2 occurred in 2 patients (1.1%) in the sentinel LND group and 11 patients (9.8%) in the radical LND group, also showing a significant difference (*p* < 0.001).

Tumor characteristics such as grade, stage, LVSI, ESMO risk, and lymph node metastases were strongly associated with increased complications (*p* < 0.001), likely due to the more radical surgeries performed on high-risk tumors. Three patients died within 30 days postoperatively, all of whom had undergone radical LND and had ACCI scores > 4.

### Oncologic Outcome

**Fig. 1:**
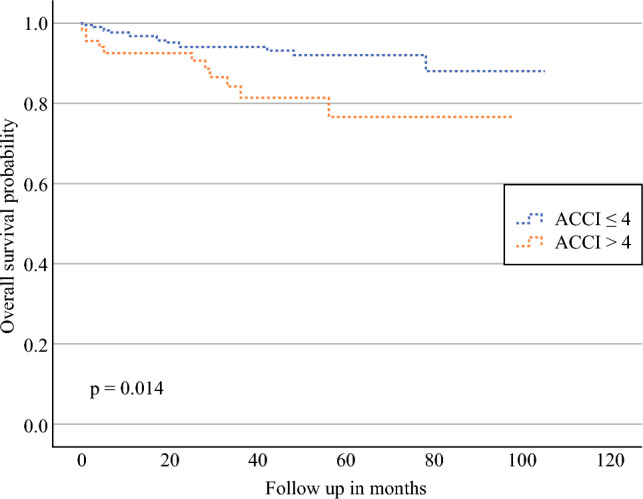
Kaplan–Meier curves comparing the OS of patients with low (≤ 4) and high (> 4) ACCI. *Note*: The *p*-value was calculated using the log-rank test. *OS* overall survival, *ACCI* Age-Adjusted Charlson Comorbidity Index

**Fig. 2:**
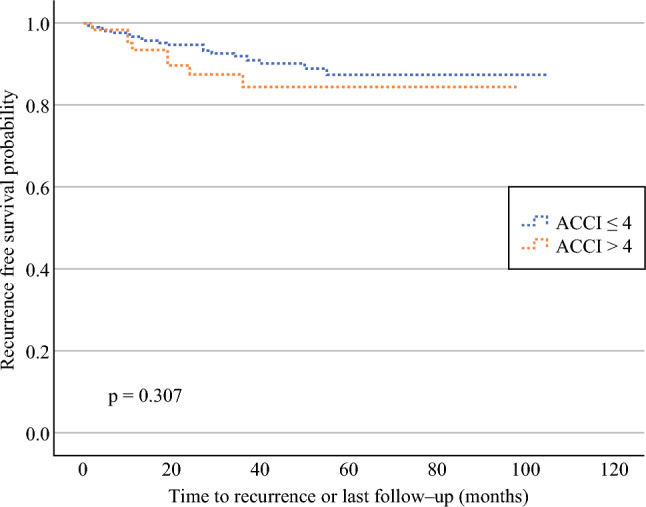
Kaplan–Meier curves comparing the RFS of patients with low (≤ 4) and high (> 4) ACCI. *Note*: The *p*-value was calculated using the log-rank test. *RFS* recurrence-free survival, *ACCI* Age-Adjusted Charlson Comorbidity Index

The univariate analysis of all parameters on RFS and OS are presented in Table 4. In the whole cohort, for frailty scores, the ACCI (> 4) showed significantly lower OS (hazard ratio [HR] 2.57, 95% CI 1.18–5.60; *p* = 0.018) (Fig. 1) but had no significant impact on RFS (Fig. 2).

**Table 4:** Univariate Cox regression analysis of parameters potentially affecting oncological outcomes

Total (sentinel LND + radical LND)					Sentinel LND only					Radical LND				
	OS		RFS		OS			RFS		OS			RFS	
	HR (95% CI)	*p*-Value	HR (95% CI)	*p*-Value	HR (95% CI)		*p*-Value	HR (95% CI)	*p*-Value	HR (95% CI)		*p*-Value	HR (95% CI)	*p*-Value
ACCI > 4	2.57 (1.18–5.60)	**0.018**	1.53 (0.67–3.51)	0.310	3.61 (1.16–11.23)		**0.027**	1.52 (0.39–5.87)	0.545	1.50 (0.53–4.28)		0.444	1.81 (0.61–5.42)	0.288
mFI > 0.27	4.22 (0.99–17.95)	0.052	0.05 (0.00–5062.49)	0.613	6.84 (1.48–31.69)		**0.014**	0.05 (0.00–388,476.31)	0.707	0.05 (0.00–4,500,071,238)		0.815	0.05 (0.00–8.91E+)	0.834
mFI-5 > 1	1.91 (0.77–4.77)	0,165	1.17 (0.41–3.40)	0.767	2.54 (0.76–8.44)		0.129	1.35 (0.29–6.36)	0.704	1.33 (0.30–5.81)		0.709	1.48 (0.33–6.61)	0.609
ASA > 2	1.62 (0.75–3.50)	0.224	1.49 (0.70–3.16)	0.304	3.00 (0.89–10.04)		0.076	2.14 (0.60–7.58)	0.239	1.21 (0.47–3.15)		0.689	1.00 (0.35–2.88)	0.998
ESMO	1.68 (1.31–2.16)	< **0.001**	1.65 (1.28–2.11)	< **0.001**	1.83 (1.22–2.74)		**0.004**	1.82 (1.17–2.94)	**0.009**	1.52 (1.01–2.27)		**0.044**	1.96 (1.22–3.15)	**0.006**
LVSI	5.90 (2.77–12.58)	< **0.001**	4.00 (1.86–8.63)	< **0.001**	11.69 (3.66–37.34)		< **0.001**	7.39 (1.88–29.05)	**0.004**	2.21 (0.85–5.74)		0.103	3.29 (1.16–9.27)	**0.016**
FIGO stage	1.32 (1.14v1.53)	< **0.001**	1.32 (1.14–1.54)	< **0.001**	1.54 (1.28–1.95)		< **0.001**	1.55 (1.13–2.12)	**0.007**	1.20 (0.99–1.46)		0.070	1.23 (1.00–1.52)	**0.049**
Grade	2.15 (1.29–3.57)	**0.002**	1.41 (0.88–2.28)	0.158	1.42 (0.60v3.36)		0.429	0.66 (0.21–2.05)	0.472	1.14 (0.56–2.34)		0.722	7.69 (1.11–53.19)	**0.039**
Histology	1.49 (1.21–1.84)	< **0.001**	1.49 (1.21–1.84)	< **0.001**	0.44 (0.00–264.39)		0.799	0.43 (0.00–587,841)	0.818	1.43 (1.13–1.80)		**0.003**	1.49 (1.17–1.91)	**0.001**
Myometrial infiltration	0.59 (0.40–0.86)	**0.006**	0.63 (0.43–0.92)	**0.017**	0.65 (0.36–1.20)		0.168	0.45 (0.24–0.83)	**0.011**	0.92 (0.57–1.49)		0.741	0.58 (0.33–1.03)	0.062
Cervical stroma invasion	0.80 (0.11–5.90)	0.826	1.59 (0.38–6.69)	0.531	0.05 (0.00–44,310.80)		0.663	2.81 (0.36–22.23)	0.327	0.88 (0.12–6.67)		0.905	1.06 (0.14–8.07)	0.955
Number of LNs+	1.17 (0.89–1.54)	**0.034**	1.47 (1.21–1.78)	< **0.001**	6.63 (0.84–52.43)		0.073	9.99 (1.22–81.73)	**0.032**	1.34 (1.08–1.66)		**0.008**	1.08 (0.79–1.49)	0.627
Age	1.07 (1.03–1.11)	< **0.001**	1.01 (0.97–1.04)	0.750	1.08 (1.03–1.14)		**0.004**	1.01 (0.96–1.07)	0.678	0.99 (0.95–1.04)		0.803	1.05 (0.99–1.11)	0.102
BMI, kg/m^2^	0.96 (0.90–1.02)	0.158	0.97 (0.92–1.03)	0.295	0.98 (0.91–1.06)		0.661	0.99 (0.92–1.07)	0.787	0.97 (0.89–1.04)		0.354	0.94 (0.85–1.03)	0.150
Chronic pulmonary disease	3.92 (1.35–11.37)	**0.012**	3.30 (0.99–10.98)	0.051	3.62 (0.79–16.35)		0.097	5.58 (1.18–26.30)	**0.030**	2.36 (0.31–17.80)		0.406	4.86 (1.08–21.76)	**0.039**
Congestive heart failure	7.66 (1.75–33.52)	**0.007**	3.87 (0.51–29.31)	0.190	0.05 (0.00–2.22E13)		0.825	0.05 (0.00–2.32E13)	0.861	5.42 (0.70–42.15)		0.107	11.16 (2.40–51.86)	**0.002**
Number of medications	1.16 (1.04–1.29)	**0.007**	0.99 (0.86–1.15)	0.900	1.24 (1.07–1.43)		**0.005**	0.98 (0.77–1.26)	0.877	1.00 (0.84–1.20)		0.991	1.10 (0.93–1.29)	0.272
Help with medication intake	1.41 (0.33–6.03)	0.642	0.66 (0.09–4.86)	0.679	3.02 (0.65–14.02)		0.158	1.96 (0.24–15.78)	0.529	0.05 (0.00–413.31)		0.505	0.05 (0.00–1793.45)	0.567
Help at home	3.99 (1.36–11.69)	**0.012**	0.80 (0.11–5.90)	0.823	9.60 (2.86–32.22)		< **0.001**	2.30 (0.29–18.46)	0.433	0.05 (0.00–992.41)		0.545	0.05 (0.00–5236.43)	0.604
Auxiliary means	1.87 (1.08–3.23)	**0.025**	1.36 (0.67–2.76)	0.397	2.34 (1.32–4.13)		**0.003**	1.83 (0.84–4.01)	0.130	0.80 (0.16–4.03)		0.783	0.85 (0.16–4.39)	0.844
Fall over the past year	2.21 (0.63–7.72)	0.214	2.22 (0.48–10.30)	0.309	3.62 (0.73–17.98)		0.116	1.51 (0.28–22.60)	0.412	2.09 (0.24–17.94)		0.501	1.25 (0.15–10.18)	0.834
Loss of weight	3.74 (1.14–12.34)	**0.029**	2.00 (0.61–6.50)	0.250	5.50 (1.09–27.67)		0.039	1.75 (0.18–16.90)	0.630	1.88 (0.47–7.55)		0.376	2.46 (0.41–14.75)	0.324
eGFR, mL/min	1.03 (1.01–1.05)	**0.013**	1.01 (0.98–1.03)	0.528	0.95 (0.92–0.98)		**0.003**	0.98 (0.98–1.02)	0.451	1.00 (0.97–1.03)		0.857	0.99 (0.96–1.03)	0.602
Hemoglobin, g/L	1.03 (1.01–1.05)	**0.001**	1.00 (0.97–1.03)	0.997	0.93 (0.86–1.00)		**0.039**	0.98 (0.94–1.02)	0.339	1.02 (0.98–1.06)		0.334	1.03 (0.98–1.08)	0.312
Albumin, g/L	1.11 (1.03–1.20)	**0.006**	0.97 (0.87–1.09)	0.590	0.91 (0.73–1.14)		0.434	1.01 (0.82–1.24)	0.945	1.04 (0.92–1.18)		0.502	1.06 (0.92–1.23)	0.413

Bold values indicate statistically significant *p*-values (*p* < 0.05)

*LND* lymph node dissection, *HR* hazard ratio, *CI* confidence interval, *OS* overall survival, *RFS* recurren *ASA* American Society of Anesthesiologists, *ACCI* Age-Adjusted Charlson Comorbidity Index, *mFI* modified Frailty Index, *mFI-5* 5-item modified Frailty Index, *ESMO* European Society for Medical Oncology, *LVSI* lymphovascular space invasion, *LNs+* positive lymph nodes, *BMI* body mass index, *eGFR* estimated glomerular filtration rate

**Fig. 3:**
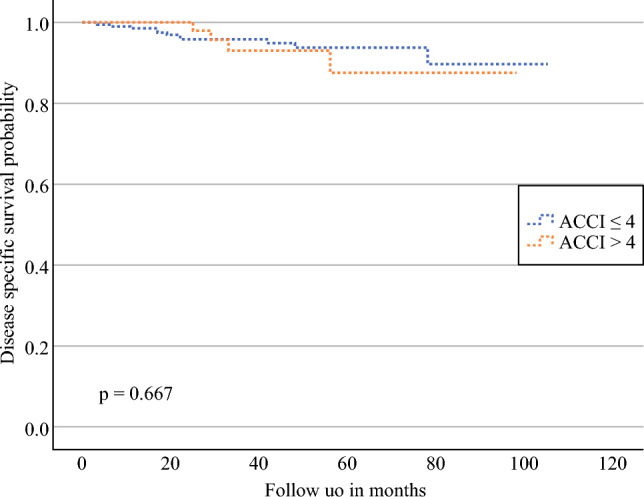
Kaplan–Meier curves comparing the DSS of patients with low (≤ 4) and high (> 4) ACCI. *Note*: The *p*-value was calculated using the log-rank test. *DSS* disease-specific survival, *ACCI* Age-Adjusted Charlson Comorbidity Index

Fifteen patients (5.2%) died from EMCA, while 12 (4.2%) died from other causes. No significant association was observed for ACCI > 4 (HR 1.29, 95% CI 0.41–4.08; *p* = 0.661). Kaplan–Meier curves for DSS stratified by ACCI score are shown in Fig. 3.

Tumor-specific parameters such as ESMO, stage, histology type, and grading are in line with what was expected, showing worse outcomes, both in OS and RFS. Of the independent variables, age, number of medications, help at home, auxiliary needs, and weight loss were associated with significantly worse OS. Comorbidities of congestive heart failure and COPD also showed significantly worse OS. The laboratory parameters showed that a lower hemoglobin, albumin, and renal function were associated with worse OS. All these parameters were significant for OS but not for recurrence.

Comparison of the same parameters between the SLN group and the radical LND group are also presented in Table 4. In the SLN group, approximately the same parameters show worse survival. A difference was observed for mFI > 0.27, also being significant for worse OS; however, COPD and congestive heart failure did not show a significant impact. Interestingly, in the radical LND group, only tumor characteristics integrated in ESMO showed a significantly worse OS.

For the multivariate analysis, the parameters were grouped by clinical setting (Table 5). For OS, ACCI, ESMO, congestive heart failure, and hemoglobin were significant risk factors, while for RFS, ESMO and congestive heart failure were significant risk factors. None of the functional parameters remained significant in multivariate analysis.

**Table 5:** Multivariable Cox regression analysis for the entire cohort (sentinel LND and radical LND combined), grouped by ACCI/ESMO, preoperative functional status, and comorbidities

	Total (SLN + rLND)					
Variable	OS			RFS		
Scores	HR	95% CI	p-value	HR	95% CI	p-value
ACCI	2.24	1.02-4.90	*0.044*	1.51	0.66-3.49	*0.330*
ESMO	1,61	1.24-2.07	< *0.001*	1.59	1.24-2.03	< *0.001*
*Functional status and age*						
Age (years)	1.08	0.97-1.19	0.162	0.91	0.82-1.01	0.067
Number of medicaments	1.16	0.78-1.75	0.466	0.99	0.66-1.48	0.949
Home assistance (home care services or family/friends)	1.14	0.14-9.47	0.902	0.00	0.00-1.275E+65	0.756
Auxiliary means	0.82	0.22-3.07	0.767	1.72×10^9	0.00-1.55 × 10^64	0.742
Fall over past year	6.93	0.67-71.57	0.104	4.46	0.01-1.49 × 10^3	0.614
Lost of weight (> 5kg over last year)	5.56	0.85-36.59	0.074	0.00	0.00-5.15 × 10^33	0.876
*Comorbidities*						
Chronic pulmonary disease	3.02	0.97-9.45	0.057	2.32	0.69-7.83	0.176
Congestive heart failure	6.29	1.35-29.27	0.019	9.36	1.06-82.49	0.044
Hemoglobin (g/L)	1.03	1.00–1.05	0.033	1.00	0.97-1.03	0.888
Albumin (g/L)	1.05	0.95–1.16	0.323	0.99	0.86–1.12	0.829
eGFR (ml/min)	1.02	0.99–1.04	0.132	1.02	0.99–1.04	0.2

*LND* lymph node dissection, *ACCI* Age-Adjusted Charlson Comorbidity Index, *ESMO* European Society for Medical Oncology, *OS* overall survival, *RFS* recurrence-free survival

## Discussion

The results of this study provide important insights into the role of frailty and its impact on postoperative outcomes and OS in patients undergoing minimally invasive surgery for EMCA. By assessing frailty through multiple scoring systems, i.e. ACCI, mFI, mFI-5, and ASA, we aimed to better understand how frailty influences clinical decisions and patient outcomes in the context of surgical management, specifically comparing sentinel LND with radical LND.

Our study found that the ACCI was the most robust predictor of postoperative complications and OS, with a cut-off of > 4 resulting in the identification of 23.2% of patients as frail. This score effectively predicted the length of hospitalization, with patients scoring > 4 experiencing a higher risk of severe postoperative complications (Clavien–Dindo > 2). The three deaths occurring within 30 days postoperatively were included in this group. Additionally, an ACCI score > 4 was associated with significantly lower OS (HR 2.57, 95% CI 1.18–5.60) and remained significant in the multivariate analysis (HR 2.24, 95% CI 1.02–4.90).

While primarily a comorbidity index, the ACCI proved more practical to apply in preoperative settings than more frailty-focused scores, and relies on information already collected during routine preoperative assessments. Studies such as those by Schipa et al.^27^ and Di Donato et al.^28^ showed similar findings in gynecologic surgery and EMCA cohorts, respectively, with lower cut-offs for ACCI (e.g., ≥ 2 or ≥ 3) predicting worse outcomes; however, those studies lacked differentiation between specific types of surgery and surgical morbidity. In our study, the ACCI stood out as the best overall predictor of poor outcomes, indicating that frail patients not only tolerated surgery poorly but also had reduced long-term survival. In our study, the ACCI emerged as the most robust overall predictor of poor outcomes, suggesting that frail patients not only experienced greater perioperative vulnerability but also exhibited reduced long-term survival. OS was selected as the primary endpoint rather than DSS to better reflect the broader impact of frailty and comorbidity in a population of women undergoing surgery for EMCA. Given the generally favorable prognosis associated with EMCA, it is not unexpected that RFS and DSS did not significantly differ across frailty strata. As anticipated, the majority of deaths in our cohort were unrelated to disease progression, highlighting the substantial role of non-cancer-related mortality in this older, comorbid population. The observed association between higher ACCI scores and poorer OS, despite no significant differences in DSS, supports the potential utility of the ACCI as a screening tool to identify patients who may be too frail for surgery or who may benefit from multidisciplinary, individualized perioperative management.

Surprisingly, in our cohort, neither the mFI nor the ASA classification significantly predicted OS or perioperative morbidity. This contrasts with studies such as the study by Pichatechaiyoot et al.^29^, which found that mFI ≥1 and ASA ≥2 were associated with higher postoperative complications; however, those studies did not account for the extent of LND, potentially introducing selection bias. Applying the widely validated mFI cut-off of 0.27 identified only 2.4% of patients in our population as frail, which may explain why this score did not predict worse outcomes in our cohort. Although an EMCA cohort typically includes patients in poor health, they often remain eligible for extensive surgeries. This could account for the low number of frail patients identified using this definition. Indeed, the 0.27 cut-off for mFI is highly selective; for example, Mogal et al. found that only 6.4% of patients undergoing pancreaticoduodenectomy were classified as frail using this threshold.^30^ However, the primary aim of these tools in our setting is not to broadly categorize patients as frail but to detect those who are truly at increased risk of adverse outcomes. Although initially developed for broader risk stratification, this targeted identification is particularly valuable in the surgical context as it enhances specificity in selecting patients who may benefit most from preoperative optimization or tailored perioperative care.

### Perioperative Morbidity

When comparing clinical factors, age, BMI, and frailty scores, we observed no significant difference between the sentinel LND and radical LND groups. This reflects the current practice of basing surgical radicality decisions on tumor characteristics rather than on patient frailty or age. This aligns with evidence that older patients generally tolerate gynecologic-oncologic surgery well and should not be excluded from optimal treatment options.^31,32^ Recent studies also support the safety and feasibility of minimally invasive surgery in elderly and even frail elderly patients, particularly with robotic approaches.^33,34^ These findings highlight the potential of a multidisciplinary approach and the need to adapt surgical radicality to individual risk profiles. Efforts to reduce morbidity remain essential, especially in vulnerable populations; however, in the era of personalized medicine, there is an increasing need to consider individual patient characteristics when planning surgical interventions.

In assessing tolerability, we confirmed the higher risk of complications in the radical LND group, consistent with existing literature.^35^ Among patients with an ACCI > 4, postoperative complications and prolonged hospitalizations were significantly more frequent. It should also be noted that in Switzerland, hospitalization times are generally longer than in other European or North American centers due to institutional policies and the structure of postoperative care.

Age > 70 years and chronic pulmonary disease emerged as significant independent predictors of both postoperative complications and OS, regardless of the extent of surgery. Patients older than 70 years of age had a higher complication risk (*p* = 0.031), while chronic pulmonary disease was uniquely associated with both worse complications (*p* = 0.013) and OS in the radical LND group. These findings emphasize the importance of thorough preoperative assessment, especially in older patients and those with respiratory comorbidities.

Chronic pulmonary disease, particularly in geriatric populations, has been linked to a nearly twofold increased risk of 30-day mortality post-surgery and may also affect oncologic outcomes via tumor microenvironment hypoxia.^36^ This aligns with findings from The Cancer Genome Atlas (TCGA) studies on hypoxia-related genetic alterations in EMCA.^37,38^ Identifying and addressing such comorbidities is critical in surgical planning and predicting outcomes.

Interestingly, laboratory markers such as hypoalbuminemia and renal function, as well as weight loss > 5%, did not predict postoperative complications despite their known association with OS. This suggests that while these parameters may reflect frailty or poor health, they are less useful in predicting immediate surgical tolerance in minimally invasive procedures. Hypoalbuminemia, for example, is a known risk factor for complications in ovarian cancer surgery but may be less relevant for smaller procedures such as hysterectomy and sentinel LND.^39^

### Oncologic Outcomes

In terms of OS, known tumor characteristics such as ESMO risk groups, histology, lymph node positivity, LVSI, and FIGO stage were predictive of both OS and RFS, consistent with previous studies.

If we look for risk factors for tumor progression, RFS or DSS are our main parameters in predicting oncological outcomes. However, since we wanted to detect risk factors that might predict outcomes for a frail patient who might not have long to live with or without EMCA, we focused on OS in looking at oncological outcomes. Studies have shown that comorbidities and more aggressive tumors are more a cumulative rather than competing risk when analyzing OS,^40^ emphasizing that identifying important comorbidities is also important to improve OS in aggressive tumors. In multivariable analysis, ACCI > 4, ESMO risk group, congestive heart failure, and hemoglobin levels remained significant predictors of OS. From all the parameters analyzed, these five factors seemed to have the most impact on OS. Surprisingly, all of these factors describe the tumor and comorbidities rather than frailty symptoms alone. Therefore, in screening for patients who might not qualify for extensive treatment, these factors are easily applicable, since they are all the usual parameters gathered before surgery. In addition, other comorbidity scores, such as the Adult Comorbidity Evaluation 27 score, have shown to be able to predict poor survival in the EMCA patient cohort.^41^ Furthermore, congestive heart failure, even adjusted by age, was shown to be an independent risk factor for perioperative morbidity and mortality. In the days of emerging pre-rehabilitation programs, future research should explore whether interventions targeting these risk factors can improve outcomes.

### Limitations and Future Directions

The retrospective design of this study introduces potential selection bias, and although validated frailty scores were used, they may not fully capture the multifaceted nature of frailty in patients with EMCA. In particular, the low number of grade 3 or higher complications in this minimally invasive surgery cohort, as well as the small proportion of frail patients according to the mFI, limits the statistical power to assess the predictive value of frailty tools, and reduces generalizability. These findings should therefore be interpreted with caution and should be considered exploratory. Moreover, distinguishing surgical morbidity from patient- or tumor-related factors remains challenging. Prospective studies are needed to validate these findings, evaluate the impact of frailty on long-term outcomes, and identify effective preoperative interventions for frail patients.

## Conclusion

In patients undergoing minimally invasive surgery for EMCA, among all the analyzed frailty factors, an ACCI score > 4 was the best predictor of perioperative morbidity, prolonged hospitalization, and reduced OS. The ACCI is a practical and effective tool for preoperative risk assessment, and scores > 4 should prompt careful consideration of the extent of surgery, especially for radical lymphadenectomy. Chronic pulmonary disease and low hemoglobin levels also warrant attention in preoperative evaluations due to their association with poor outcomes.

Incorporating frailty assessments into standard preoperative protocols, alongside molecular tumor profiling, could enhance personalized treatment planning. Future research should focus on prospective trials to refine frailty metrics, identify modifiable preoperative risk factors, and explore targeted interventions to improve outcomes for frail patients.
